# Pitfalls of Adrenal Washout on CT in the Adult Population: A Pictorial Review

**DOI:** 10.3390/diagnostics16060920

**Published:** 2026-03-19

**Authors:** Benjamin Hao Bai Tay, Jordan Zheng Ting Sim

**Affiliations:** Department of Diagnostic Radiology, Tan Tock Seng Hospital, 11 Jalan Tan Tock Seng, Singapore 308433, Singapore

**Keywords:** adrenal nodule, adrenal washout, hypervascular adrenal nodule, phaeochromocytoma, hypervascular adrenal metastasis, lipid-poor adrenal adenoma, periadrenal varix

## Abstract

Adrenal washout CT (AWCT) is a well-established reference standard for differentiating adenomas from non-adenomas and has been widely adopted in clinical practice for over three decades. However, in recent years, studies have demonstrated the low specificity of these washout values in diagnosing adenomas, with the latest 2023 European Society of Endocrinology guidelines noting the low quality of evidence regarding the accuracy of these cutoff values. Phaeochromocytomas are recognised as the classic mimickers of adenomas on AWCT as they exceed conventional washout thresholds. However, radiologists should be familiar with other hypervascular lesions to avoid potential diagnostic pitfalls. These hypervascular entities can pose a diagnostic challenge to radiologists who rely too much on washout values and percentages. In this pictorial essay, a comprehensive range of hypervascular adrenal lesions are illustrated. Incorporating lesion morphology, ancillary features, biochemical markers and the presence of extra-adrenal disease are essential for an accurate diagnosis and for avoiding inappropriate management.

## 1. Introduction

Adrenal nodules are common incidental findings detected on cross-sectional imaging occurring in approximately 5% of scans [1,2]. Computed tomography (CT) is the most common imaging method used in diagnosis of adrenal lesions due to its widespread use and high spatial resolution. A multiphasic acquisition protocol helps to achieve the diagnosis, especially in cases of diagnostic uncertainty, with a dedicated adrenals protocol typically consisting of three phase acquisitions: non-contrast, portal venous phase (obtained at 60 s) and delayed phase (obtained at 15 min) [3].

Historically, adrenal washout CT (AWCT) has been regarded as the reference standard for differentiating adenomas from non-adenomas and has been widely adopted in clinical practice for over three decades [4]. These AWCT values are derived via calculation of the absolute percentage washout (APW), $\left[\frac{HUpv-HUdl}{HUpv-HUnon}\right]\times 100$, and relative percentage washout (RPW) $\left[\frac{HUpv-HUdl}{HUpv}\right]\times 100$, with HU = Hounsfield units, HUpv = HU on portal venous phase, HUdl = HU on delayed phase, and HUnon = HU on non-contrast enhanced phase. Adenomas typically show an APW value of 60% or greater and an RPW value of 40% or greater, which have a 98% sensitivity and 92% specificity for diagnosis [3]. However, these previously established washout values are not specific to adrenal adenomas.

The June 2023 clinical practice guidelines published by the European Society of Endocrinology opined that while a cutoff of 60% for APW and 40% for RPW are often taken as diagnostic for adrenal adenomas, the level of evidence backing the accuracy of these cutoff values for the incidentaloma population is low [5]. A recent review article by Seow et al. also argued for the elimination of AWCT to avoid over-investigation in the incidentaloma population [4].

A retrospective cross-sectional study by van Aswegen et al. in 2024 described the sensitivity of the reported absolute washout to be 33% and the specificity to be 77% [6]. Schloetelburg et al. in 2021 also showed that these established cutoffs of APW and RPW incorrectly identified up to 22% of malignancies [7]. Up to a third of phaeochromocytomas are also known to demonstrate similar washout characteristics [4,8,9].

The objective of this work is to offer a concise pictorial review of the lesions that can show washout on AWCTs and provide reasonable differential diagnoses. In this review, we present a range of hypervascular adrenal lesions that can demonstrate contrast enhancement characteristics that cross the APW and RPW thresholds. These lesions range from malignant to benign pathologies, including phaeochromocytomas, hypervascular metastases, adrenocortical carcinomas, lipid-poor adenomas, retroperitoneal haemangiomas, periadrenal varices and adrenal haemorrhage.

While phaeochromocytomas, hypervascular metastases and adrenocortical carcinomas can show overlapping features on CT, specific key features such as history of primary malignancy or biochemical markers can aid in differentiation. Retroperitoneal haemangiomas typically show heterogeneous progressive enhancement with centripetal filling, though they often have non-specific imaging features. Adrenal haemorrhage is also a known mimicker of masses and can demonstrate varying features. Varices located in close proximity to the adrenal gland can often mimic a hypervascular adrenal mass requiring close scrutiny on multiple planar reformatting. While the focus of this paper lies in hypervascular adrenal lesion mimics with similar washout patterns to adrenal adenomas, establishing the classic radiological profile of an adrenal adenoma is also essential for understanding how diagnostic pitfalls occur.

## 2. Phaeochromocytoma

Phaeochromocytomas are rare catecholamine-secreting neuroendocrine tumours arising most frequently from the chromaffin cells of the adrenal medulla. While these are most frequently diagnosed in people in their 40s and 50s, they can affect individuals of any age. Most of these tumours are biochemically active, they are also commonly diagnosed incidentally on imaging and account for up to 3–5% of adrenal incidentalomas [10]. About two in five phaeochromocytomas are hereditary, more than any other type of endocrine tumour [11]. Among the known hereditary conditions, the most common are those of multiple endocrine neoplasia type 2A (MEN2A), type 2B (MEN2B), von Hippel–Lindau syndrome type 2 (VHL 2) and mutations in succinate dehydrogenase B, C and D [11,12].

Diagnosis of phaeochromocytoma (PCC) is based on a combination of clinical features and biochemical testing, with subsequent imaging for tumour localisation [13,14]. The initial biochemical testing should include elevated plasma catecholamine levels and elevated 24 h urinary catecholamine metabolites [13,14,15,16]. Other biochemical markers may include urinary dopamine and plasma 3-methoxytyramine when testing for tumours with high risk for metastases or predominant dopamine secretion and chromogranin A for biochemically silent tumours [14,17]. Patients with phaeochromocytomas usually show signs and symptoms of excess catecholamines, with the triad of headache, palpitations, and sweating being 90% specific [18]. Other, less specific signs can include fever, flushing, nausea, loss of weight, fatigue, anxiety symptoms, uncontrollable high blood pressure, orthostatic hypotension, and elevated blood glucose [10,11,19].

**Figure 1:**
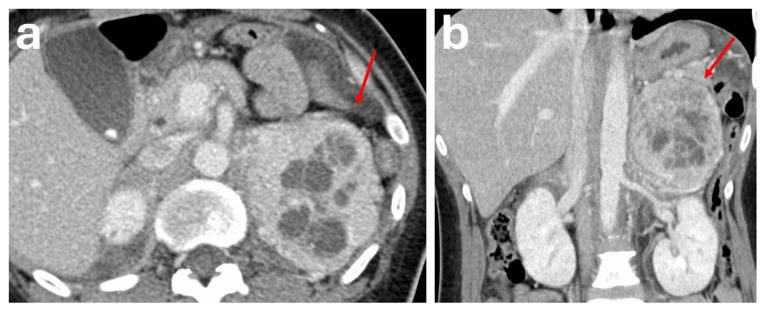
Patient with a history of hypertension and unexplained weight loss over a 12-month period had a CT TAP performed for malignancy screening. The CT demonstrated a large 9.0 × 8.7 × 7.0 cm heterogeneous mass in the left suprarenal region (red arrow): (**a**) axial plane, (**b**) coronal plane. The solid areas of the mass showed avid enhancement on the portal venous phase (HU 140). Internal areas of non-enhancement represent necrosis. The mass displaced the adjacent pancreatic tail and splenic flexure with no evidence of invasion. Biochemical testing showed raised urine and plasma metanephrines. This was proven to be a phaeochromocytoma post-resection. Generic testing showed a pathogenic variant in the MAX gene (MYC-associated factor X), associated with hereditary paraganglioma–phaeochromocytoma (PGL-PCC) syndrome.

CT is advised for initial work-up due to its high spatial resolution when phaeochromocytoma is suspected biochemically [13,14]. However, imaging diagnosis of phaeochromocytoma on CT is often difficult due to variable imaging characteristics [11,20,21,22]. They typically appear as a heterogeneous, avidly enhancing mass ranging from 4 to 6 cm. The smaller lesions tend to demonstrate homogeneous enhancement, whereas larger lesions tend to demonstrate heterogeneous appearances with central hypodensity due to necrosis, cystic change (Figure 1) or haemorrhage [18,19,23]. Calcifications are common and can be seen in approximately 10% of cases and in up to 21% of symptomatic phaeochromocytomas [24]. The solid enhancing components of these masses will usually demonstrate an internal attenuation exceeding 85 HU after administration of contrast on the portal venous phase [25]. Northcutt et al. suggested that a 130 HU threshold is 100% specific for identifying phaeochromocytoma [26]. Although avid enhancement is a key feature seen in most phaeochromocytomas, it is not specific to phaeochromocytomas and may be seen in other types of vascular adrenal lesions. Furthermore, all phaeochromocytomas have metastatic potential, with a reported rate of 10.2% of cases developing metachronous or metastatic disease during follow-up [19]. At times, larger masses or metastatic phaeochromocytomas may exert mass effect, demonstrate invasion of adjacent structures or demonstrate tumour thrombus, making it difficult to differentiate them from adrenocortical carcinoma [11,23,27]. These lesions also demonstrate inconsistent washout patterns on adrenal CT protocol, with articles showing that up to one-third of phaeochromocytomas demonstrate washout characteristics such as adenomas on delayed CT scans and could be confused with adenomas [4,8,9,11].

MRI is an alternative imaging modality and is usually preferred when CT findings are inconclusive [14] or for specific groups—for example, children, pregnant women and patients who require frequent follow-up imaging—to reduce or prevent ionising radiation exposure [28]. On MRI, these lesions classically show a “light bulb sign”, with a homogeneous high T2w signal in approximately two-thirds of cases [28]. Positron emission tomography with computed tomography (PET/CT) or single-photon emission computed tomography (SPECT)/scintigraphy is the preferred functional imaging modality, usually performed for patients with known metastasis or multiplicity [13,28]. The most common radionuclides are 123I-MIBG, 18F-FDG, 68Ga-DOTA-somatostatin analogues (SSAs) and 18F-FDOPA [14].

Surgical resection is the mainstay of therapy. It can be either a minimally invasive adrenalectomy (e.g., laparoscopic) or open resection [13], with pre-operative initiation of α-adrenergic receptor blockers to reduce the risk of hypertensive crisis and cardiovascular complications [13,29,30]. Patients with metastatic phaeochromocytoma may also benefit from adjunct chemotherapy or radionuclide therapy [11,17,18,23]. Biopsy is contraindicated due to the risk of releasing excess catecholamine levels and causing a life-threatening hypertensive crisis [29,30].

Post-operative follow-up measurements of patients’ plasma or urine levels of metanephrines should be obtained to evaluate for recurrence [13], as up to 6% of patients develop recurrent or metastatic disease [31]. Post-operative imaging is mainly done to detect residual or recurrent tumours when biochemical tests are positive [28]. All patients diagnosed with phaeochromocytoma should also be recommended for genetic testing due to a large proportion of cases linked to inherited mutations [13].

## 3. Lipid-Poor Adrenal Adenoma

Adenomas are the most common benign adrenal neoplasms, representing 50–80% of adrenal lesions [21,22,32]. Their incidence increases with age, with most of them being non-functional, and they are detected incidentally. Functioning adenomas can produce excess glucocorticoids leading to Cushing’s syndrome or mild autonomous cortisol secretion (MACS), mineralocorticoids leading to hyperaldosteronism, and, at times, androgens leading to androgen excess syndrome [5,32].

They are typically well-circumscribed, rounded, homogenous masses measuring 1–4 cm in size, demonstrating stability or slow interval growth on follow-up imaging with a lack of aggressive features such as invasion of adjacent organs or metastasis [5,20,33].

**Figure 2:**
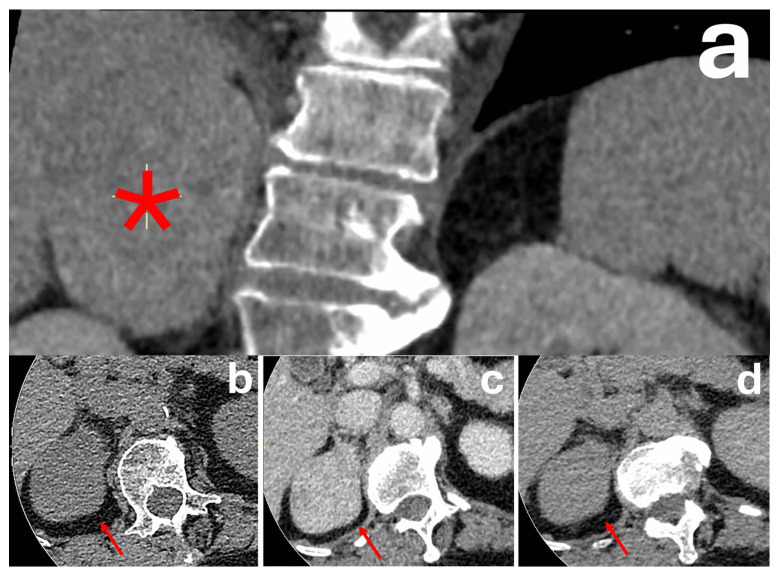
Patient with an incidental right suprarenal mass (red asterisk) measuring 7.4 cm in size with displacement of the right kidney (**a**). The mass is well circumscribed and homogeneous with no evidence of invasion of adjacent structures. It measures (red arrows) 32 HU on the pre-contrast phase (**b**), 121 HU on the portal venous phase (**c**), and 53 HU on the delayed phase (**d**). Absolute washout was 76.4%. Relative washout was 56.2%. Surgical resection was performed due to the size of the mass and raised 24 h urinary free cortisol, suggesting hypercortisolism. Histopathology was consistent with an adrenal cortical adenoma.

Most of them contain abundant intracytoplasmic fat and demonstrate a HU < 10 on unenhanced CT, which is a highly diagnostic feature of lipid-rich adenomas [34,35]. Approximately 30% of adenomas are lipid-poor and demonstrate higher attenuation values on unenhanced HU [34]. While diagnosis of lipid-poor adenomas was historically based on AWCT washout values due to their rapid enhancement and washout characteristics, these are no longer reliable due to their low specificity (Figure 2) [4,5,6,7]. As the size of the adenomas increase, they can at times demonstrate heterogeneous appearances with necrosis/cystic change, haemorrhage or calcification, which may make it difficult to differentiate them from malignant lesions such as hypervascular metastasis, phaeochromocytoma, or adrenocortical carcinoma [20,23].

MRI is an alternative diagnostic modality and is used mainly to differentiate adenomas from non-adenomas. As adenomas typically contain inherent fat, chemical shift MRI (CSI) sequences are typically used to look for intralesional fat, seen as a loss of signal intensity from the in-phase to the out-phase [20,33,36].

Functioning adrenal adenomas are typically managed with surgical resection, whereas non-functioning adenomas with indeterminate CT features require further evaluation and continued surveillance [37].

## 4. Adrenocortical Carcinoma

Adrenocortical carcinomas (ACCs) are rare, aggressive adrenal tumours arising from the adrenal cortex [17]. They have a bimodal age distribution, peaking in early childhood and middle adulthood [38,39]. While they are most commonly sporadic, they can be associated with hereditary conditions such as Li-Fraumeni syndrome, familial adenomatous polyposis coli, MEN-1, Carney complex, Beckwith–Wiedemann syndrome and Lynch syndrome [5,21,40].

Most ACCs are secretory, with excess adrenal hormone secretion seen in up to 60% of cases and patients presenting with symptoms of hormonal excess. A third present with nonspecific symptoms, whereas the rest (20–30%) are incidentally diagnosed [40]. Based on the ESMO–EURACAN Clinical Practice Guidelines 2020, all suspected ACCs require extensive endocrine work-up for identification of autonomous excess of glucocorticoids, sex hormones, mineralocorticoids and adrenocortical steroid hormone precursors [17]. This includes a 1 mg dexamethasone suppression test (DST), an aldosterone/renin ratio assessment, and testing for free cortisol in 24 h urine, basal plasma adrenocorticotropic hormone (ACTH), dehydroepiandrostenedione sulfate, 17-hydroxyprogesterone, androstenedione, testosterone (women only), 17-beta-oestradiol (men and postmenopausal women only), and serum potassium. Phaeochromocytoma should also always be ruled out via testing of plasma catecholamine levels and 24 h urinary catecholamine metabolites [17].

**Figure 3:**
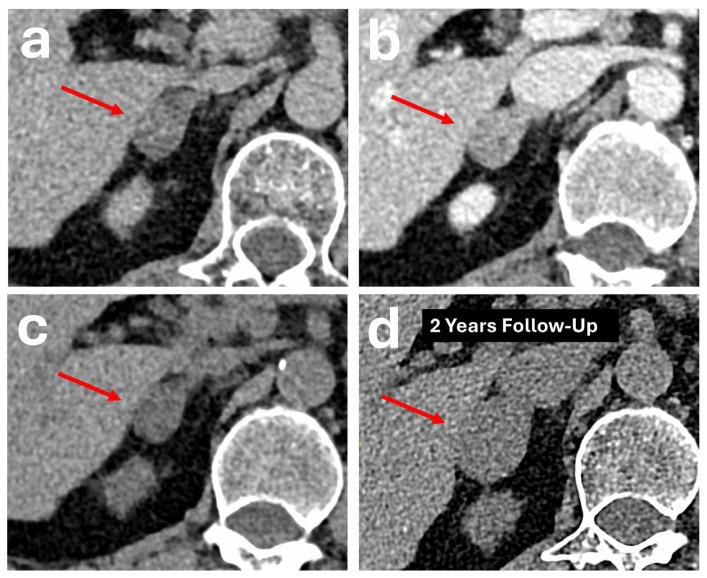
Patient with an incidentally diagnosed, well-circumscribed, homogeneous 2.2 × 1.3 cm nodule arising from the lateral limb of the right adrenal gland (red arrows). It measures 20 HU on pre-contrast (**a**), 83 HU on the portal venous phase (**b**), and 43 HU on the delayed phase (**c**). Absolute washout was 63.5%. Relative washout was 48.2%. This was initially diagnosed as a lipid-poor adrenal adenoma due to its washout values. Follow-up unenhanced CT performed 2 years later showed an increase in nodule size to 2.9 × 2.3 cm (red arrow). No aggressive features were noted (**d**). The patient showed no abnormal biochemical markers. Surgical resection was performed with histopathology revealing an adrenocortical adenocarcinoma.

On CT, ACCs usually present as a large heterogeneously enhancing mass measuring more than 5 cm [27]. Post-contrast, they enhance heterogeneously, with central areas of non-enhancement due to haemorrhage or necrosis [20,23,38]. Calcification is also a common finding, and it is usually central in location, seen in approximately 30% of patients with ACC. In contrast, this finding is rare in adenomas and is observed in only about 10% of phaeochromocytomas [38]. In a few instances, intratumoural macroscopic fat has been noted due to the presence of fat within the cytoplasm of adrenal cortical cells. Hence, sole reliance of intratumoural fat on imaging is not sufficient to differentiate between adenomas and ACC [23,27,38]. They typically show an APW of less than 60%, although there have been a few studies reporting high washout values similar to those of adenomas (Figure 3) [23]. Other features that suggest malignancy include locoregional invasion of adjacent structures such as the kidneys and liver, venous invasion into the adjacent renal vein, and inferior vena cava or tumour thrombus. Metastatic spread is another feature of malignancy, seen in approximately 30% of patients, which can be seen as nodal spread to the adjacent retroperitoneal lymph nodes or distant metastasis to the liver, lungs, bones or peritoneum [6,23,27,38].

MRI as an alternative imaging technique is known to be equally as effective as CT. FDG-PET/CT is occasionally used, especially if there is suspicion of bone metastasis [17].

Surgery is the primary curative option for ACC, with open surgery being the standard treatment approach [17]. As recurrence is common, systemic mitotane therapy is often used in adults. Adjuvant therapy or radiotherapy may be considered on a case-by-case basis [17]. Of note, radiotherapy is contraindicated in patients with Li-Fraumeni syndrome due to a potential increase in susceptibility to radiation-associated malignancies [41]. Chemotherapy is also used in cases with advanced or metastatic disease [17]. Following a total resection, intensive radiological monitoring and clinical and biochemical testing are also essential to monitor for recurrence [17,39].

## 5. Hypervascular Metastasis

The adrenal glands represent a frequent site for secondary malignancy; in patients with a pre-existing cancer diagnosis, 30% to 70% of incidentally detected adrenal masses are confirmed as metastases [42]. Furthermore, general population autopsy data indicates an adrenal metastasis prevalence of 3.1% [43].

Most adrenal metastases demonstrate unenhanced attenuation values of >10 HU with heterogeneous appearance and, often, rapid growth [42]. While many adrenal metastases do not demonstrate significant enhancement, hypervascular adrenal metastases are known to occur in hypervascular extra-adrenal primary malignancies, such as renal cell carcinoma (RCC) [44] and hepatocellular carcinoma (HCC) [45], with up to 95% of cases meeting adenoma APW criteria [46].

**Figure 4:**
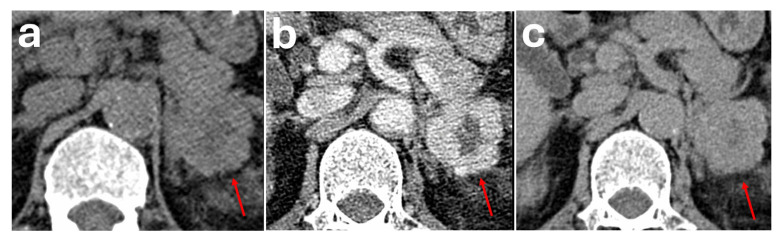

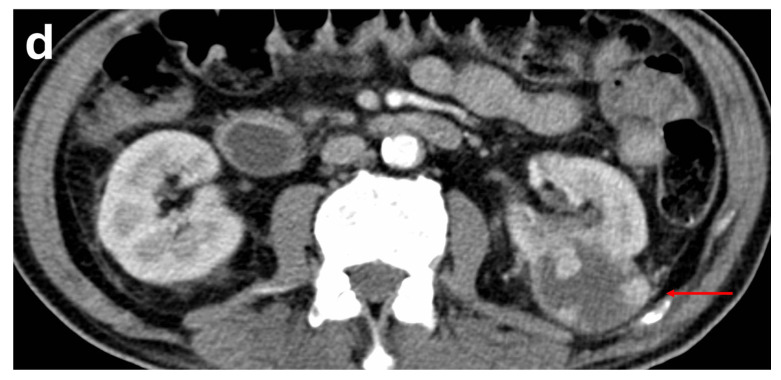
Patient with a CT demonstrating an avidly enhancing, lobulated mass in the left adrenal gland body with central necrosis (red arrows) measuring 4.3 × 3.8 cm. It shows 28 HU on the pre-contrast phase (**a**), 112 HU on the portal venous phase (**b**), and 54 HU on the delayed phase (**c**). Absolute washout was 69.0%, and the relative washout was 51.8%. Imaging through the rest of the abdomen reveals an exophytic solid-cystic mass in the left kidney midpole (red arrow), compatible with a primary renal malignancy (**d**). Biochemical markers were normal. Surgical resection of the left renal and adrenal masses confirmed the presence of renal cell carcinoma with metastasis of the left adrenal gland.

**Figure 5:**
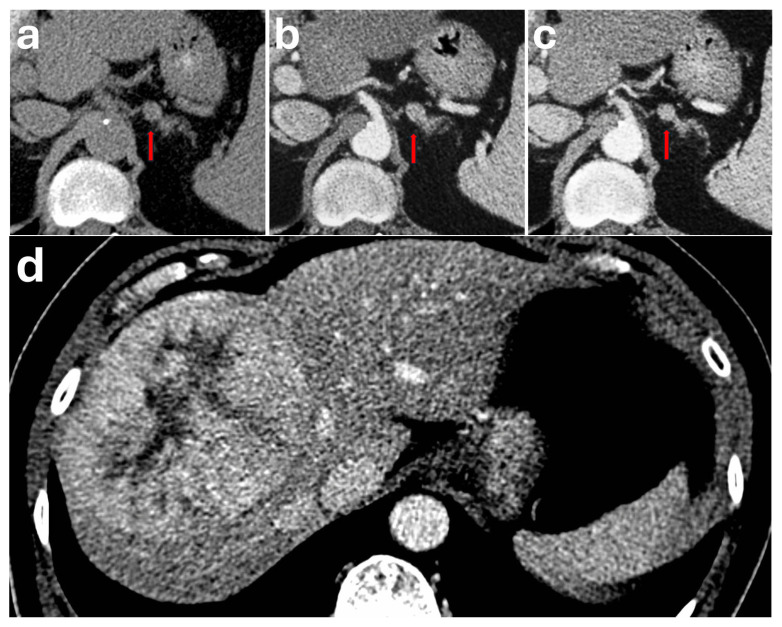
Patient with a prior surgical history of hepatic VII segmentectomy for hepatocellular carcinoma (HCC) presented with rising alpha-fetoprotein levels. CT demonstrated a 1.4 cm homogeneously enhancing nodule arising from the body of the left adrenal gland (red arrows). It demonstrated 44 HU on the pre-contrast phase (**a**), 105 HU on the portal venous phase (**b**), and 58 HU on the delayed phase (**c**). Absolute washout was 77.0% and relative washout was 44.8%. Biochemical markers were normal. Subsequent resection of the left adrenal gland confirmed metastasis from HCC. (**d**) Initial CT scan demonstrating the primary hepatocellular carcinoma localised to segment VII of the liver. There was no evidence of distant metastasis to the adrenal glands on the initial scan.

These hypervascular adrenal metastatic masses typically demonstrate a high attenuation of > 140 HU on the portal venous phase and rapid washout on the delayed phase, as one would expect to see in a typical RCC or HCC (Figure 4 and Figure 5, respectively) [20,46]. In these scenarios, relying purely on APW/RPW percentages can lead to misdiagnosis of metastases as benign adenomas.

Often, differentiating between an ACC and metastasis based on imaging alone may be impossible. In patients with a history of known primary malignancy, the detection of an adrenal mass with atypical features should raise suspicion of metastasis in the reader [23]. In a retrospective study by Suntornlohanakul et al., 96.2% of patients with diagnosed adrenal metastasis had a known diagnosis of a primary malignancy at the time of referral [47]. The presence of bilateral adrenal masses is also a clue and is reported in 43% of cases [48].

Although infrequent, an occult malignancy may metastasize to the adrenal gland and present as a solitary mass on initial presentation [49]. In such scenarios, close interval follow-up, targeted investigations for a primary malignancy and/or histopathological confirmation may be considered.

## 6. Retroperitoneal Haemangioma

Retroperitoneal haemangiomas are rare benign vascular tumours, with a few cases reported in the literature, most often of the cavernous haemangioma subtype [50,51,52,53]. These lesions are seen in the retroperitoneal cavity, typically in or around the adrenals, kidneys, urinary tract, or pancreas [51], or within the retroperitoneal fat [52]. Typically affecting older populations [53], these lesions are often asymptomatic at discovery. However, larger masses can cause mass effect and compress on surrounding structures, leading to non-specific symptoms like back pain, haematuria, or lower urinary tract symptoms [51,53].

**Figure 6:**
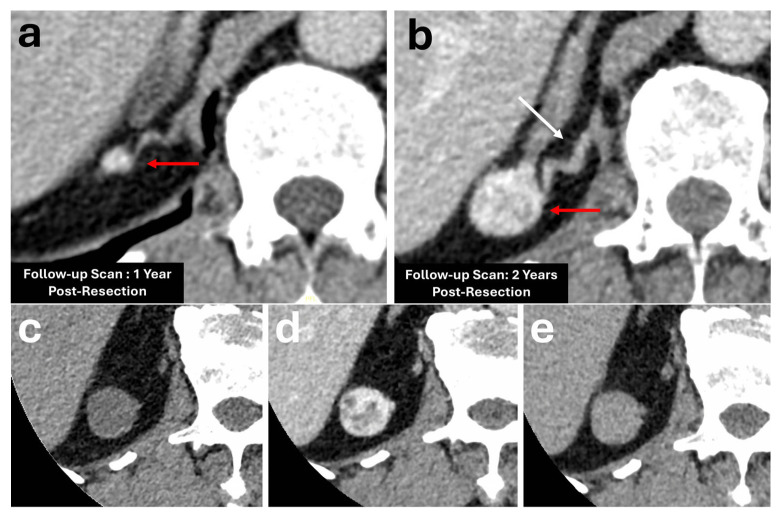
Patient with prior surgical resection of a functioning left adrenal adenoma. Follow-up CT examinations post-resection (**a**,**b**) show a gradually enlarging heterogeneous hypervascular nodule (red arrows) closely related to the right adrenal gland measuring 2.4 × 2.3 cm (previously 0.9 cm). A feeding vessel was noted on the anteromedial aspect of the lesion (white arrow). The nodule was 22 HU on the pre-contrast phase (**c**), 125 HU on the portal venous phase (**d**), and 70 HU on the delayed phase (**e**). Absolute washout was 53.4%. Relative washout was 44.0%. The patient remained asymptomatic with normal biochemical markers. Surgical resection was performed showing fibroadipose tissue with lobular aggregates of dilated blood vessels lined by bland endothelial cells. Mature adipose tissue and areas of hyalinisation were noted within the tumour. Findings were consistent with a haemangioma.

These lesions are typically described as well-defined, low-density lesions with heterogeneous progressive enhancement (Figure 6), with centripetal filling and high peak values of enhancement on the venous phase reaching above 150 HU [50,53]. However, these lesions are also described as having varying non-specific imaging features with heterogeneous features and areas of non-enhancement due to degeneration, intravascular thrombosis or haemorrhage [50,51].

While these lesions are benign with a favourable prognosis, the range of imaging findings makes them difficult to diagnose without histopathological correlation. The diagnosis of retroperitoneal haemangioma should be considered if a lesion shows typical characteristics of avid enhancement with centripetal filling, which can help to prevent unnecessary surgical intervention [53].

## 7. Periadrenal Varix

Various retroperitoneal pathologies may be misidentified as hypervascular adrenal masses, particularly when they abut or maintain close contact with the adrenal glands [20]. Periadrenal varices frequently result from portal hypertension or prominent feeder vessels to renal and pancreatic neoplasms, as well as prominent diaphragmatic crura, and they can often mimic adrenal tumours through their anatomical proximity [54]. While a clinical history of liver cirrhosis leading to portal hypertension may clue us into its diagnosis, patients are usually asymptomatic with no known liver disease [55].

These vascular lesions are most often seen in the left periadrenal region anterior to the adrenal gland, where there are often splenorenal and gastrorenal shunts in patients with portal hypertension, and in patients in whom there are prominent vessels in the inferior phrenic-adrenal-renal venous pathway [54,55]. Anatomically, the left adrenal vein joins the left inferior phrenic vein, which traverses anterior to the adrenal gland and drains into the left renal vein [56].

**Figure 7:**
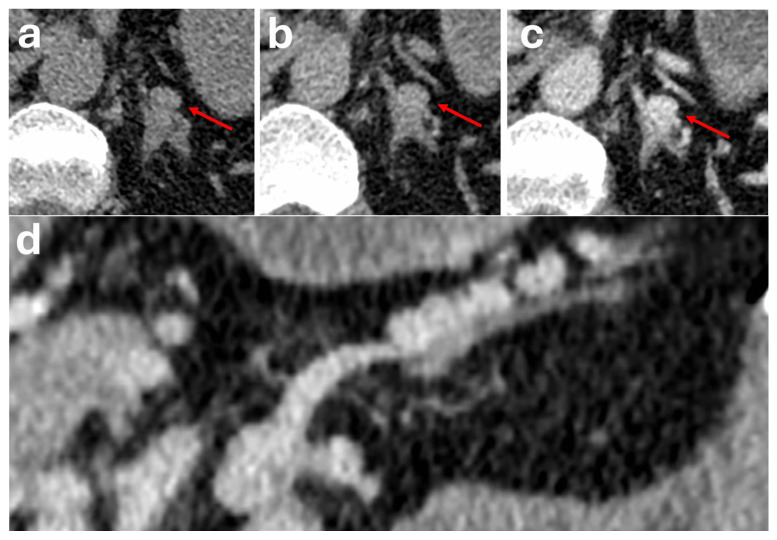
Patient with an incidental 1.3 cm left adrenal nodule measuring 44 HU on unenhanced CT thorax (red arrows) (**a**). The nodule measured 145 HU on portal venous phase (**b**) and 65 HU on delayed phase (**c**). Absolute washout is 79.2%. Relative washout is 55.2%. On multiplanar reformat, the nodule was noted to be part of a serpiginous structure that enhanced in tandem with adjacent vessels and demonstrates continuation with a tributary of the left renal vein (**d**). Overall features are in keeping with an adrenal varix. The patient remained asymptomatic with no evidence of liver cirrhosis and a normal liver Fibroscan result.

On unenhanced CT, they may mimic an ovoid, homogeneous soft-tissue adrenal mass (Figure 7). After the administration of intravenous contrast, the extra-adrenal location of these varices and their vascular nature will become more apparent, demonstrating a serpiginous appearance and enhancing in tandem with adjacent vessels [54,55].

It is critical to avoid confusing a periadrenal varix with an adrenal tumour, as an accidental biopsy or excision could have dire consequences leading to retroperitoneal haemorrhage [20]. Reviewing a lesion across multiple planes and using multiplanar reformatted (MPR) sequences will help in the precise localisation of these masses. Particularly in patients with known chronic liver disease or portal hypertension, recognising prominent periadrenal vascular collaterals or varices is always important so that they are not misidentified as primary adrenal tumours.

## 8. Adrenal Haemorrhage

**Figure 8:**
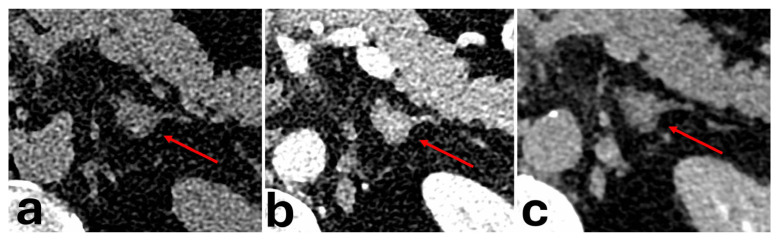
Patient with a background of newly diagnosed hepatocellular carcinoma with a 1.3 cm left adrenal nodule detected on liver CT. Dedicated adrenal CT was performed to rule out adrenal metastasis. The nodule (red arrows) shows 19 HU on the pre-contrast phase (**a**), 125 HU on the portal venous phase (**b**), and 44 HU on the delayed phase (**c**). Absolute washout was 76.4%. Relative washout was 64.8%. AWCT values could not differentiate between a hypervascular metastasis and an adrenal adenoma. Patient remained asymptomatic with normal biochemical markers. Biopsy was subsequently performed with histopathology findings of dilated blood vessels with haemorrhage.

A broad spectrum of benign adrenal pathologies can mimic the appearance of malignant hypervascular masses, frequently posing a diagnostic dilemma on multiphasic CT due to their indeterminate appearances. Adrenal haemorrhage (AH) is a notable mimic, with its heterogeneous and complex appearance easily mistaken for an adrenal neoplasm (Figure 8) [57]. In addition to trauma, stress, and coagulopathy, AH may present as an intratumoral bleed or coexist alongside other glandular pathologies [57,58]. For this reason, clinicians should maintain a high suspicion for underlying malignancy in any patient presenting with non-traumatic adrenal haemorrhage [59,60].

Patients tend to demonstrate non-specific symptoms, most often depending on the size of the haematoma, severity of bleeding and extent of adrenal tissue involvement. Signs and symptoms include abdominal or back pain, nausea, vomiting, haemorrhagic shock, confusion, fever, or a decrease in haemoglobin levels [60]. Adrenal insufficiency may also be present in cases with severe bilateral adrenal haemorrhage [57,59].

While they have highly variable CT imaging features, acute AH typically present as an oval or round nodule with high attenuation values on unenhanced sequences of 50–90 Hounsfield units and can have a large variance in size from a few millimetres to 10 cm [57,58,59]. Occasionally, early AH have been shown to demonstrate a “train-track appearance” due to thick and peripherally enhancing adrenal tissue around a central hypodensity, resembling parallel railroad tracks. This may also be associated with periadrenal fat-stranding [60]. A hallmark feature of AH is its progressive reduction in size and HU over time, eventually undergoing either complete resolution or evolving into a chronic organised haematoma, described as a hypoattenuating central core [57,59].

However, there are few described cases when AH presents as a mass with solid enhancing peripheral components with central fluid density [57,58,61]. This was proposed to be due to organisation and endothelial proliferation, which is more evident within the peripheries of a haemorrhagic lesion [61]. This makes differentiation between an adrenal haemorrhage and other malignant masses with solid-cystic appearances difficult due to the overlap in features, for example, phaeochromocytoma, ACC, or hypervascular metastasis [57,61].

MRI can be a useful aid in diagnosis, with the presence of high T1w signals and/or susceptibility artefacts on gradient-recalled echo (GRE) sequences to suggest blood products in the adrenals [58]. Detailed staging of the age of the blood products can also be made by identifying the haemoglobin breakdown products and their characteristic appearances on T1- and T2-weighted images [60].

## 9. Discussion

### 9.1. Approach to Hypervascular Lesions

Due to the wide range of hypervascular adrenal lesions, which can demonstrate washout, it is important to have a structured framework for approaching these lesions from a diagnostic point of view.

Assessing an adrenal lesion in multiple planes or on multiplanar sequence reformatting helps to rule out a periadrenal lesion (e.g., periadrenal varices) mimicking an adrenal mass. Retroperitoneal haemangiomas show variable imaging characteristics with non-specific clinical features, making it difficult to diagnose. At times, the classic pattern of centripetal filling and progressive enhancement (if present) may clue you into the diagnosis, although histopathological correlation may often be required for differentiation. Features of malignancy such as locoregional invasion, tumour thrombus, or the presence of metastasis should prompt a diagnosis of a tumour with malignant potential. A known primary malignancy (especially HCC or RCC in the context of hypervascular masses) or the presence of bilateral adrenal tumours should prompt the differential of adrenal metastasis. While differentiation between a malignant/large phaeochromocytoma or adrenocortical carcinoma may be difficult on imaging, clinical presentation and biochemical testing are essential with elevated plasma/urinary metanephrines seen in phaeochromocytomas and the majority of ACCs showing excess steroid secretion (e.g., glucocorticoids, sex hormones, mineralocorticoids and adrenocortical steroid hormone precursors).

The table below summarises the clinical presentation, biochemical markers, CT imaging features and alternative imaging modalities for each individual lesion (Table 1).

### 9.2. Alternative Imaging Modalities

Currently, there is no single imaging modality that is able to distinguish malignant from benign adrenal masses. CT is usually the first imaging tool for diagnosing adrenal lesions due to its high spatial resolution. Adrenal incidentalomas are also most commonly picked up on CT [1,2].

MRI is helpful in cases where the adrenal mass shows indeterminate/non-conclusive findings, or in instances where there are contraindications to CT or usage of iodinated contrast media [14].

PET/CT is a functional imaging modality for patients with known metastasis or multifocal disease [13,28]. 18F-FDG (PET/CT) is highly sensitive for differentiating malignant from benign adrenal tumours and staging aggressive cancers due to malignant tumours having higher glucose metabolism than benign tumours [62]. However, it has lower specificity and is unable to differentiate between different types of malignant disease (such as metastasis vs. phaeochromocytoma vs. adrenocortical carcinoma) [11,62].

Alternative functional imaging modalities include 18 F-FDOPA PET/CT, 123 I-MIBG SPECT/scintigraphy, and 68 Ga-DOTA-somatostatin analogues (SSAs), which are highly specific for diagnosing or staging phaeochromocytomas [11,14].

### 9.3. Current Recommendations

In view of the poor sensitivity of washout values, there needs to be a shift from over-reliance on these values. Instead, they should be used as an adjunct to other imaging features along with patient’s clinical presentation and evidence of excess hormonal secretions to aid diagnosis and management.

The June 2023 clinical practice guidelines published by the European Society of Endocrinology propose further management and follow-up for all adrenal masses based on features that reflect malignancy risk, such as heterogeneity, tumour size and unenhanced HU [5]. They recommend that if non-contrast CT shows features of a benign adrenal adenoma (homogeneous and ≤10 HU), no further imaging is needed. If CT shows a homogeneous adrenal mass that is less than 4 cm with an unenhanced HU 11–20 and no significant hormone excess on work-up, follow-up imaging in 1 year is advised. If the adrenal mass is more than 4 cm and heterogeneous or has an unenhanced HU of more than 20, there is a relevant risk for malignancy, which will require further work-up. The guidelines also recommend that all adrenal incidentalomas should be evaluated for signs and symptoms of adrenal hormone excess [5].

## 10. Conclusions

Both benign and malignant hypervascular masses of the adrenal glands can show “washout” on AWCT, thus mimicking an adrenal adenoma; hence the importance of knowledge and familiarity with these pitfalls to prevent misdiagnosis. This article shares the common key features of these hypervascular lesions and proposes a framework for evaluation of these lesions. Despite best efforts, histological confirmation or resection may still be required to establish a definitive diagnosis if imaging proves inconclusive.

## Figures and Tables

**Table 1:** Key features of hypervascular adrenal lesions.

	Clinical Presentation/Biochemical Markers (If Any)	Key CT Imaging Features	Alternative Imaging/Confirmatory Studies
**Phaeochromocytoma**	Signs and symptoms of excess catecholamines. Triad of **headache, palpitations, sweating (highly specific)** Germline mutations in 40% (e.g., MEN2A, MEN2B, VHL 2, succinate dehydrogenase B, C and D mutations) **Elevated plasma catecholamine and 24 h urinary catecholamine metabolites (highly specific)**	Avidly enhancing mass **(often >130 HU)**; homogeneous enhancement if small; heterogeneous appearance if large with necrosis/cystic/haemorrhagic change. If malignant, there may be local invasion, tumour thrombus or metastasis.	MRI if CT is inconclusive or patient deemed not suitable **PET/CT** or **SPECT** for evaluation of metastasis or multiplicity.
**Lipid-Poor Adenoma**	Majority are asymptomatic and non-functioning. Some can secrete steroid hormones	Well-circumscribed, rounded, homogenous mass measuring 1–4 cm, **lack of aggressive features**. Demonstrates stability on follow-up, rapid washout on AWCT (though non-specific). If large, it can show heterogeneous appearance with necrosis/cystic change, haemorrhage or calcification.	MRI to look for inherent fat
**Adrenocortical Carcinoma (ACC)**	**Features of excess steroid hormone in up to 60%**	Large (>5 cm); heterogeneous mass due to haemorrhage or necrosis; may show central calcification (30%). Can show intratumoural macroscopic fat Features of malignancy: locoregional invasion of adjacent structures, venous invasion, tumour thrombus or metastatic spread	MRI is an alternative equally as effective as CT. FDG-PET/CT can be used, especially if there is suspicion of bone metastasis
**Hypervascular Metastasis**	**History of primary cancer (e.g., RCC, HCC)**	High attenuation of >140 HU, **often bilateral (43%).** Can show rapid growth and clinical history of extra-adrenal malignancy.	Histopathological correlation or correlation with known primary malignancy
**Retroperitoneal Haemangioma**	Non-specific presentation	Non-specific features, although **classically show centripetal filling and progressive enhancement** reaching >150 HU on venous phase	At times, histopathological correlation is required due to non-specific features
**Periadrenal Varix**	Non-specific presentation. Patients may have history of chronic liver disease leading to **portal hypertension**	Ovoid lesion mimicking a hypervascular adrenal mass if close to the adrenal gland. Often requires MPR to show its **periadrenal location**. Typically showing a **serpiginous appearance** and **enhancing in tandem with vessels** (often showing continuation with renal/phrenic veins	Nil specific
**Adrenal Haemorrhage**	Non-specific presentation. Common aetiologies: **trauma, stress, coagulopathy**, underlying adrenal lesion	Highly variable features. Typically demonstrates high unenhanced HU (50–90 HU), **temporal evolution is a hallmark feature** (reduction in size and HU on follow-up)	Serial follow-up CT imaging showing resolution or size reduction

## Data Availability

No new data were created or analysed in this study. Data sharing is not applicable to this article.
